# Three-dimensional image reconstruction of distribution of Pnmt^+^ cell-derived cells in murine heart

**DOI:** 10.1038/sdata.2017.134

**Published:** 2017-09-26

**Authors:** Haibo Ni, Yange Wang, William Crawford, Shanzhuo Zhang, Longxian Cheng, Henggui Zhang, Ming Lei

**Affiliations:** 1 School of Physics and Astronomy, University of Manchester, Manchester M13, 9PL, UK; 2 Key Laboratory of Medical Electrophysiology of Ministry of Education, Collaborative Innovation Center for Prevention and Treatment of Cardiovascular Disease/Institute of Cardiovascular Research, Southwest Medical University, Luzhou 6400, China; 3 Department of Pharmacology, University of Oxford, Oxford OX1 3QT, UK; 4 Department of Cardiovascular Disease, Union Hospital, Tongji Medical College of Huazhong University of Science and Technology, Wuhan 430022, China

**Keywords:** Optogenetics, Cardiovascular biology

## Abstract

Elucidating the function of specific cell types in a highly complex multicellular system such as the heart often requires detailed anatomic reconstruction. We recently described a distinctive class of phenylethanolamine n-methyltransferase (Pnmt^+^) cell-derived cardiomyocytes (PdCMs), a new cardiomyocyte population with a potential endocrine role. In this dataset, a 3D reconstruction was carried out to visualise the distribution of PdCMs throughout the murine heart. Rigid registration (stiff rotation and translation) was applied to properly align the fused heart slice images based on landmarks using TrakEM2, an open source plug-in in Fiji. The registered slices were then analysed and reconstructed using MATLAB (MATLAB^®^. *Version 8.3.0.532)*. The final reconstructed 3D volume was 561×866×48 pixels (corresponding to spatial resolutions of 5.8, 8.9 and 2.5 mm in the x-, y- and z-direction respectively), and visualised in Paraview. The reconstruction allows for detailed analyses of morphology, projections and cellular features of different cell types, enabling further geometrical and topological analyses. Image data can be accessed and viewed through *Figshare*.

## Background & Summary

A major challenge faced in biomedical research is to define the function of specific cell types in a highly complex multicellular system. A detailed anatomical reconstruction of their distribution will no doubt facilitate such research. In our recent study^1^, we identified Pnmt^+^ cell-derived cardiomyocytes (PdCMs) in the murine heart by introducing channelrhodopsin 2 (ChR2) specifically into murine cells expressing the *Phenylethanolamine n-methyltransferase* (*Pnmt*) gene, which encodes the enzyme responsible for conversion of noradrenaline to adrenaline. This murine model (Pnmt-Cre/ChR2 mice) enabled us to identify a distinctive class of Pnmt-expressing neuroendocrine cells and their descendants (i.e. Pnmt^+^ cell-derived cells) within the heart. We showed that Pnmt^+^ cell-derived cells are predominantly localized to the left side of the adult heart^1^. Remarkably, we found many of the Pnmt^+^ cell-derived cells in the left atrium and ventricle appeared to be working cardiomyocytes based on their morphological appearance and functional properties^1^. These PdCMs are similar to conventional myocytes in morphological, electrical and contractile properties^1^. By stimulating PdCMs selectively with blue light (470nm wavelength, 2 ms duration, generated by a Transistor-transistor logic (TTL) -controlled light emitting diode (LED)), we were able to control cardiac rhythm in the whole heart, isolated tissue preparations and single cardiomyocytes. Thus our new murine model effectively demonstrates functional dissection of cardiomyocyte subpopulations using optogenetics, and opens new frontiers of exploration into their physiological roles in normal heart function as well as pathological roles in disease heart^1^.

**Figure 1:**
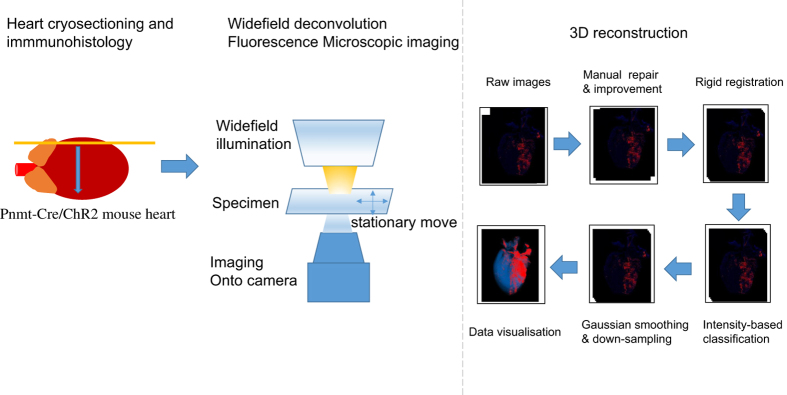
A schematic overview of the workflow of the study. Left panel: the processes of heart cryosectioning and immmunohistology. Middle: imaging process. Fluorescence images for detecting ChR2/tdTomato positive cells were obtained using Olympus FV1000 Confocal or Zippy Moving-stage Fluorescent microscopes. Right: diagram illustrating the procedures of the 3D reconstruction. Raw images were first manually improved and registered; an intensity-based classification was applied to obtain the positively stained cells; reconstructed 3D volume data was visualised in Paraview following Gaussian smoothing and down-sampling.

Given the potentially undiscovered roles of PdCMs, a detailed examination of their distribution in the heart with anatomical reconstruction is necessary. To achieve this goal, a comprehensive dataset has been generated by experimentation and computation using the Pnmt-Cre/ChR2 mouse heart model. These mice express an improved channelrhodopsin-2/tdTomato fusion protein in Pnmt^+^ cell-derived cells, which includes PdCMs. Figure 1 illustrates a schematic overview of the study design.

**Figure 2:**
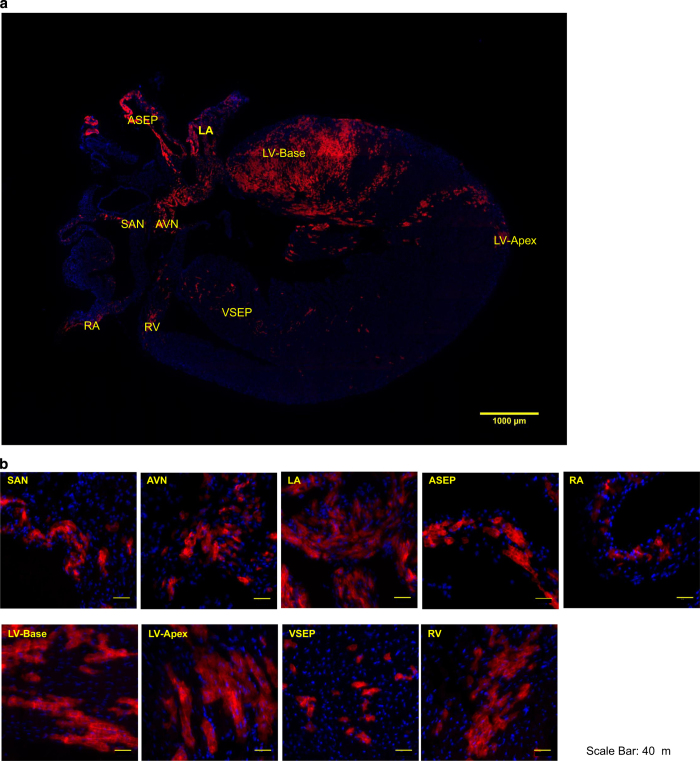
Representative images of the coronal section and selected regions from the section of an adult ChR2/tdTomato mouse heart showing fluorescence and morphology of the ChR2/tdTomato positive cells. Representative images of the coronal section and selected regions from the section of an adult ChR2/tdTomato mouse heart showing fluorescence and morphology of the ChR2/tdTomato positive cells. (**a**) A representative coronal section from an adult ChR2/tdTomato mouse heart; (**b**) inserts of zoom-in views showing tdTomato fluorescence in different regions of the heart; the labelling of the inserts indicates the corresponding locations as marked in **a**. AVN: atrioventricular node; ASEP: atrial septum; LA: left atrium; LV: left ventricle; SAN: sinoatrial node; VSEP: ventricular septum; RA, right atrium, RV: right ventricle.

**Figure 3:**
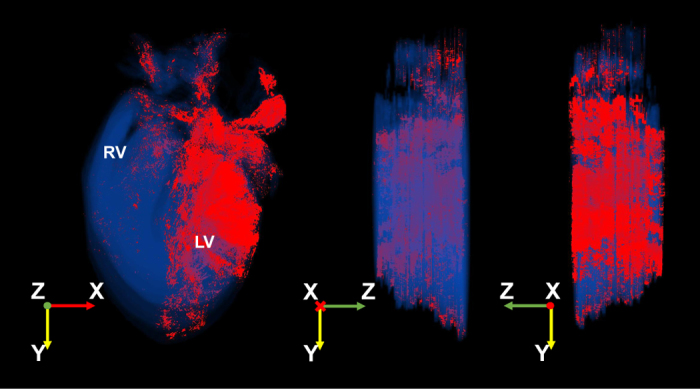
3D reconstruction of the distribution of ChR2/tdTomato positive cells in different regions of the heart using customised computer programs and scripts to generate 3D representations of the ChR2/tdTomato staining patterns. Multiple views are given.

The dataset has numerous advantageous and unique features. Firstly, the conditional expression of Pnmt-Cre/ChR2 facilitates expression of the useful ChR2/tdTomato fusion protein in a tissue-specific manner. The ChR2 light-sensitive subunit of this fusion protein causes depolarisation and contraction of cardiomyocytes with stimulation by blue light, while imaging is facilitated by the tdTomato fluorescent protein. Secondly, imaging of the series of fixed tissue sections from Pnmt-Cre/ChR2 mouse hearts was performed using wide-field deconvolution fluorescence microscopy. This technique produces high quality digital images equivalent to confocal images with high contrast and resolution, but with low fluoresce light. The concurrent use of a programmed moving stage allowed for the capture of up to one hundred images from a single tissue section, which was assembled into a two-dimensional (2D) coronal section images using softWoRx (Scientific Imaging, Seattle, USA) for further reconstruction as presented in Fig. 2. Thirdly, three-dimensional (3D) reconstruction (Fig. 3, Data Citation 1: Online Video 1) was carried out for improved anatomical visualization. Rigid registration (stiff rotation and translation) was applied to align the stitched heart slice images based on landmarks using the TrakEM2 (ref. 2), an open source plug-in in Fiji^3^. The registered slices were then reconstructed into a single file in VTK format using MATLAB (MATLAB^®^, *Version 8.3.0.532)*. The final reconstructed 3D volume was 561×866×48 pixels, and visualised using Paraview (http://www.paraview.org/), an open source application for data analysis and visualisation. The reconstruction allows for detailed analyses of morphology, projections and cellular features of different cell types. The high density of reconstructions enables geometrical and topological analyses.

The dataset and 3D reconstructed model presented here offer a means for reuse and a basis for further development of functional models of the heart by incorporating physiological data in the future.

## Methods

Most of the methods have been described in detail in our previous publication^1^.

The mouse line was generated by crossing Pnmt-Cre mice^4^ with B6.Cg-*Gt (ROSA)26Sor*^*tm27.1(CAG-COP4H134R/tdTomato) Hze*^/J strain (Stock No. 012567, Jackson Labs)^5^.

### Immunohistology and imaging

All mice were sacrificed via cervical dislocation in accordance to the Animals Scientific Procedures Act (1986). The heart was isolated and cannulated at the aorta to allow retrograde perfusion of solution. Phosphate-buffered saline (PBS) was flushed through the heart to remove any residual blood. Adrenal glands were also isolated to serve as positive controls. Isolated tissues required for immunohistochemistry were embedded in Optimal Cutting Temperature (OCT) compound and frozen with isopentane in dry ice for 30 min. 10 μm coronal or transverse sections were then taken using a Leica CM 3050S cryostat, which were then mounted on Polylysine coated microscope slides.

After mounting of specimens, all slides were washed in PBS and fixed with 4% paraformaldehyde (PFA) (Sigma-Aldrich). Slides were again washed with PBS, mounted in Vectashield mounting medium (Vector Labs) and sealed with a coverslip and nail varnish. Fluorescence images were obtained using Olympus FV1000 Confocal and Zippy Moving-stage Fluorescent microscopes. Antibodies for immunostaining of Adrenal gland sections were applied as follows: 1) primary antibody: Anti-Pnmt (monoclonal rabbit, Abcam, UK) was used as 1:50; 2) secondary antibody: Goat anti-rabbit secondary antibody (Invitrogen, UK) was used as 1:1,000 dilutions.

### 3D Image Reconstruction

Raw images capturing fractional block of heart sections were stitched using softWoRx (Scientific Imaging, Seattle, USA). The stitched images (16-bit TIFF) were first adjusted using the ‘Auto-Contrast’ tool in ImageJ^6^ to increase the contrast between the regions with positive/negative staining and non-tissue background. The high contrasted images were visually inspected and saved as JPEG images (8-bit) to facilitate manual removal of artefacts and segmentation, as well as reducing the computational cost. A total number of 48 images capturing complete sections of the heart were selected and used in the reconstruction. The selected images were then manually repaired to remove background light contamination and noise arising from the imaging procedures. In order to reduce the computational cost in image registration, the RGB images were converted to grey scale images using MATLAB: the positively stained regions (defined based on the intensity of the red channel with a threshold of 20 to 30 for different slices, which was determined based on visual inspections) were marked by adding 100 to the intensity. Down-sampling was performed with Gaussian smooth to further reduce the size of the images. Semi-automatic rigid registration (stiff rotation and translation) was subsequently carried out to align the heart slices based on landmarks using TrackEM2 (ref. 2), an open source plug-in to Fiji^3^. Nearest-neighbour interpolation was then implemented for the image slices in which the intensity of the background tissue was too small to distinguish. The pixel-wise staining was classified by the intensity level of the pixel based on the histograms of the images: pixels with intensity over 80 were considered to be positively stained, between 3 and 80 as negatively stained, and below 3 to be non-tissue background. The image volume was subsequently processed with region growing method^7^ to remove the background noises outside the heart. The resulting data was then written to data file of VTK format (structured points (http://www.vtk.org/)) for visualisation. A down sampling in both X and Y dimension was necessary to reduce the size of the VTK file to facilitate fast visualisation on a typical office desktop (with Intel(R) Core(TM) i5-2400 CPU 8GB RAM). The reconstructed 3D volume was 561×866×48 pixels (corresponding to 5.8, 8.9 and 2.5 mm in the x-, y- and z-direction respectively). The resultant data file was examined and visualised in Paraview (http://www.paraview.org/), an open source application for data analysis and visualisation.

3D reconstruction of biological tissue using histological images has been implemented extensively^2,8,9^. In a previous study, Osuala *et al.*^9^ showed a distinctive distribution of adrenergic-derived cells in murine heart through 3D reconstruction of stained histological images. Csepe *et al.*^8^ performed 3D reconstruction of human sinoatrial node using histological sections of the region. In 3D reconstruction of histological images, image registration has to be performed in order to align the images that are often misaligned along the z-stacks. The affine transformation, a non-rigid image registration method, was implemented by Csepe *et al.*^8^. Similar to our study, Osuala *et al.*^9^ applied rigid registration in aligning the slices of the heart. The open source software TrakEM2 presented by Cardona *et al.*^2^ is a valuable platform for image volume composition from 2D histological images and provides key functionalities such as data management and semi-automatic/automatic image registration on affordable computers. Therefore, in the present study TrakEM2 was used to perform the alignment of the images of the heart sections. Although the present study focuses on reporting a dataset showing a 3D mapping of the distribution of PdCMs in the murine heart, the applied reconstruction method was shown to be cost-effective while capable of achieving desirable quality of 3D reconstruction. Our method was similar to but had advantages in simplicity over previous methods used to perform 3D reconstruction.

### Code Availability

MATLAB scripts writing image stacks into a VTK file are provided accompanying the raw images (The ‘Script’ folder in the Data Citation 1). Instructions and comments can be found within individual scripts.

## Data Records

The datasets have been made available from *Figshare* (Data Citation 1).

The datasets consist of stitched raw images, analysed images, a data file of reconstructed 3D volume representing the distribution of ChR2/tdTomato positive cells in the mouse heart, and scripts used in reconstructing the 3D volume representation from the processed images.

### Raw datasets: fluoresce microscopic image stack

‘Stitched Raw Images 1’ (Data Citation 1) contains 27 serial 2D coronal section images with strong negative staining background in JPEG format covering the Pnmt-Cre/ChR2 mouse heart. Images have been acquired, stitched and aligned as described in Methods.

‘Stitched Raw Images 2’ (Data Citation 1) contains 21 serial 2D coronal section images with weak negative staining background in JPEG format covering the Pnmt-Cre/ChR2 mouse heart. Images have been acquired, stitched and aligned as described in Methods.

### Processed datasets: analysed image stack and 3D data file

‘Processed Images’ (Data Citation 1) contains analysed, registered and down-sampled images, which were subsequently used in 3D reconstruction.

‘Visualisation’ (Data Citation 1) contains VTK files representing the reconstructed 3D distribution of the TdTomato positive cells in the mouse heart, and can be visualised using Paraview. An instruction to data visualisation is also given.

‘Script’ (Data Citation 1) consists of MATLAB scripts used to reconstruct a 3D mouse heart from the analysed images as well as a sample output using the scripts.

Online Video 1 shows a video of the reconstructed 3D heart representing the distribution of the TdTomato positive cells in the mouse heart. A summary of these datasets is given in Table 1.

## Technical Validation

**Figure 4:**
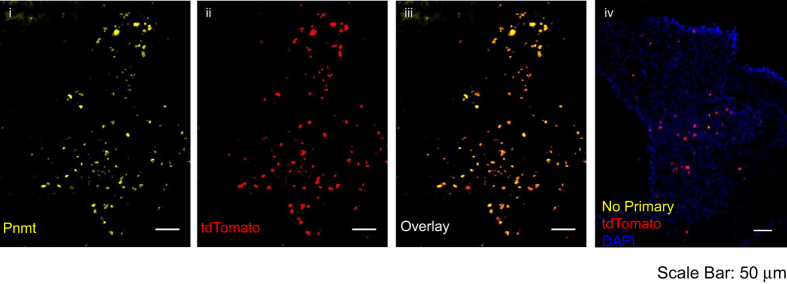
Pnmt staining and tdTomato fluorescence in adrenal medulla tissue. (i) Immunostaining of *Pnmt* with anti-Pnmt antibody; (ii) tdTomato fluorescence in a representative section of *Pnmt*^*Cre/ChR2*^ adrenal medulla; (iii) overlay of i and ii, showing the co-localisation of Pnmt and tdTomato fluorescence; (iv) without Pnmt staining.

Validation of the genotype of mice used was performed to confirm the accuracy of subsequent results. Mice were genotyped using PCR to detect the presence of the Cre-recombinase gene (forward primer CCATCTGCCACCAGCCAG; reverse primer TCGCCATCTTCCAGCAGG). Hearts from mice identified as Pnmt^+/+^ R26R^ChR2-tdTomato/ChR2-tdTomato^ were imaged to determine the presence of tdTomato fluorescence (see Fig. 4). No fluorescence was detected in this wavelength.

Further validation of the model was performed by immunohistochemistry on Pnmt^Cre/+^ R26R^ChR2-tdTomato/ChR2-tdTomato^ adrenal medulla tissue. Tissues were stained with anti-Pnmt primary antibody, and imaged for Pnmt and tdTomato expression. Fig. 4 shows identical expression of Pnmt and tdTomato, demonstrating that tdTomato expression is localized to cells that have expressed Pnmt.

## Usage Notes

A VTK file incorporating the image volume is given in the ‘Visualisation’ folder of the online data repository (Data Citation 1). The visualisation of the reconstructed volume was performed using Paraview (http://www.paraview.org/, version 5.2.0). A detailed instruction on data visualisation using Paraview is given in ‘Visualisation/Guide for Visualization.docx’ (Data Citation 1).

## Additional information

**How to cite this article**: Ni, H. *et al.* Three-dimensional image reconstruction of distribution of Pnmt^+^ cell-derived cells in murine heart. *Sci. Data* 4:170134 doi: 10.1038/sdata.2017.134 (2017).

**Publisher ’s note**: Springer Nature remains neutral with regard to jurisdictional claims in published maps and institutional affiliations.

## Supplementary Material



## Figures and Tables

**Table 1:** A summary of files available from the online data repository (Data Citation 1).

Name	Description	Format
Stitched Raw Images 1	27 serial 2D coronal section images with strong negative staining background covering the Pnmt-Cre/ChR2 mouse heart.	JPEG
Stitched Raw Images 2	21 serial 2D coronal section images with weak negative staining background covering the Pnmt-Cre/ChR2 mouse heart.	JPEG
Processed Images	The stitched raw images were analysed and rigidly registered. The pixels in the images were classified as positive/negative staining based on the intensity. Down-sampling was applied multiple times to reduce the size of the images.	JPEG
Visualisation	A VTK file containing the 3D reconstructed representation of the distribution of the ChR2/tdTomato positive cells in the mouse heart. Data can be visualised using Paraview. A configuration file (PVSM format) for visualisation and an instruction on usage are given.	VTK, PVSM
Script	MATLAB scripts used to reconstruct the 3D representation of the mouse heart from the processed images.	MATLAB script
Online Video 1	Video illustrating the 3D reconstructed distribution of ChR2/tdTomato positive cells in the murine heart.	AVI
